# Construct validity of the OCTOPuS stratification algorithm for allocating patients with knee osteoarthritis into subgroups

**DOI:** 10.1186/s12891-021-04485-1

**Published:** 2021-07-21

**Authors:** Jesper Knoop, Raymond W. J. G. Ostelo, Martin van der Esch, Arjan de Zwart, Kim L. Bennell, Marike van der Leeden, Joost Dekker

**Affiliations:** 1 Department of Health Sciences, VU University Amsterdam, De Boelelaan 1105, 1081 Amsterdam, HV Netherlands; 2 Department of Epidemiology and Data Science, Amsterdam UMC, Location VUmc, Amsterdam, Netherlands; 3 Amsterdam Rehabilitation Research Center, Reade, Amsterdam, Netherlands; 4 Center of Expertise Urban Vitality, Health Faculty, Amsterdam University of Applied Sciences, Amsterdam, Netherlands; 5 School of Health Sciences, Department of Physiotherapy, University of Melbourne, Melbourne, Australia; 6 Department of Rehabilitation Medicine, Amsterdam UMC, Location VUmc, Amsterdam, Netherlands

**Keywords:** Phenotypes, Stratification, Construct validity, Knee osteoarthritis, Exercise therapy

## Abstract

**Background:**

We recently developed a model of stratified exercise therapy, consisting of (i) a stratification algorithm allocating patients with knee osteoarthritis (OA) into one of the three subgroups (‘high muscle strength subgroup’ representing a post-traumatic phenotype, ‘low muscle strength subgroup’ representing an age-induced phenotype, and ‘obesity subgroup’ representing a metabolic phenotype) and (ii) subgroup-specific exercise therapy. In the present study, we aimed to test the construct validity of this algorithm.

**Methods:**

Data from five studies (four exercise therapy trial cohorts and one cross-sectional cohort) were used to test the construct validity of our algorithm by 63 a priori formulated hypotheses regarding three research questions: (i) are the proportions of patients in each subgroup similar across cohorts? (15 hypotheses); (ii) are the characteristics of each of the subgroups in line with their proposed underlying phenotypes? (30 hypotheses); (iii) are the effects of usual exercise therapy in the 3 subgroups in line with the proposed effect sizes? (18 hypotheses).

**Results:**

Baseline data from a total of 1211 patients with knee OA were analyzed for the first and second research question, and follow-up data from 584 patients who were part of an exercise therapy arm within a trial for the third research question. In total, the vast majority (73%) of the hypotheses were confirmed. Regarding our first research question, we found similar proportions in each of the three subgroups across cohorts, especially for three cohorts. Regarding our second research question, subgroup characteristics were almost completely in line with the proposed underlying phenotypes. Regarding our third research question, usual exercise therapy resulted in similar, medium to large effect sizes for knee pain and physical function for all three subgroups.

**Conclusion:**

We found mixed results regarding the construct validity of our stratification algorithm. On the one hand, it is a valid instrument to consistently allocate patients into subgroups that aligned our hypotheses. On the other hand, in contrast to our hypotheses, subgroups did not differ substantially in effects of usual exercise therapy. An ongoing trial will assess whether this algorithm accompanied by subgroup-specific exercise therapy improves clinical and economic outcomes.

**Supplementary Information:**

The online version contains supplementary material available at 10.1186/s12891-021-04485-1.

## Introduction

Knee osteoarthritis (OA) is a chronic joint disease that is characterized by large variability in etiology, onset, course, and treatment response among patients [1]. To better understand the disease and its treatment, the knee OA population may need to be classified into multiple (homogeneous) phenotypes or subgroups of patients. Identifying homogenous, clinically relevant subgroups could result in more effective, tailored treatments, thereby optimizing clinical and economic outcomes [2, 3]. In a previous study, we identified five homogeneous subgroups from a large knee OA cohort, based on only a small number of easily obtainable patient characteristics (i.e., body mass index (BMI), quadriceps muscle strength, depression and radiographic severity) [4]. Subsequently, we were able to replicate this finding in another large clinical cohort [5], which supports the possible existence of these subgroups. These subgroups probably correspond with the knee OA phenotypes proposed by Bijlsma et al. [1] (i.e., ‘post-traumatic’, ‘age-induced’ and ‘metabolic’ phenotypes), and might be helpful for tailoring recommended core treatments in knee OA, such as exercise therapy [6]. A tailored, subgroup-specific approach of exercise therapy could optimize the modest effects of exercise therapy on knee pain and physical function in patients with knee OA [7].

**Fig. 1:**
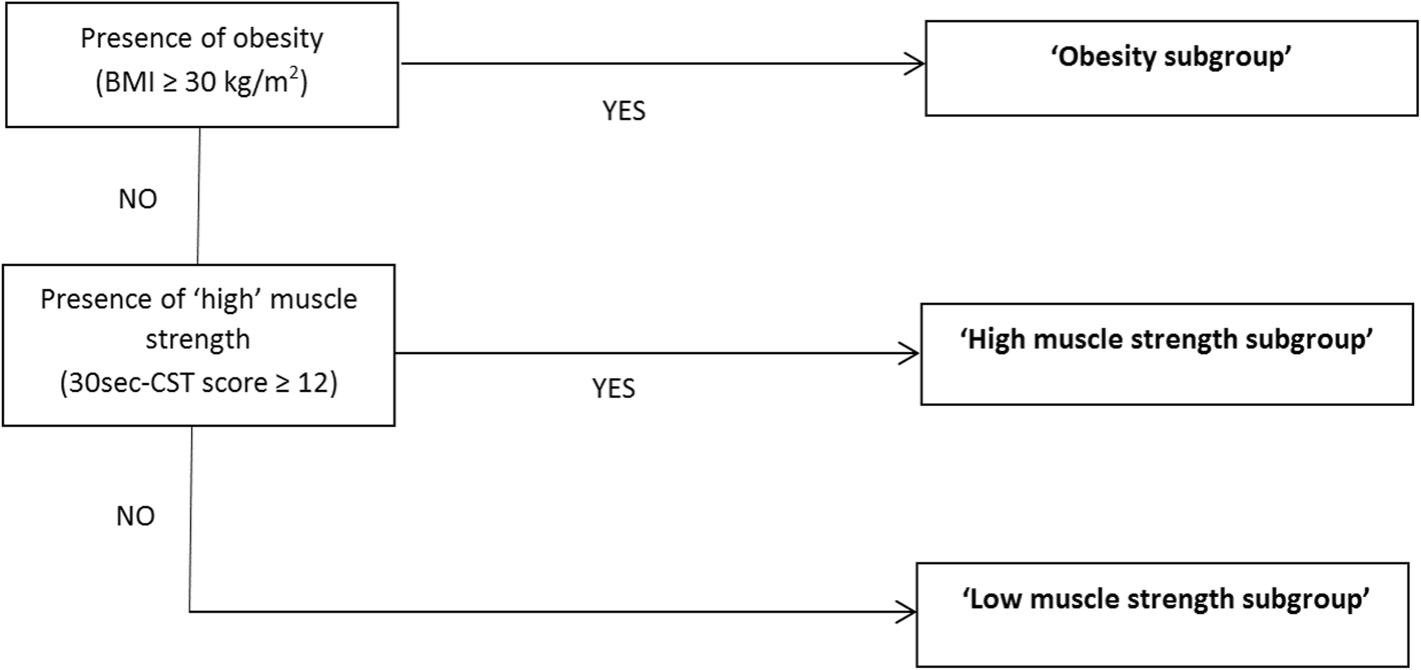
Stratification algorithm. BMI = body mass index; 30s-CST = 30 s chair stand test; rep. = repetition

Based on our phenotype identification, we developed a stratified care model, consisting of (i) a stratification algorithm (see Fig. 1) that allocates patients into subgroups by BMI and upper leg muscle strength, which are the two most clinically relevant, modifiable and easily obtainable patient characteristics in knee OA [8–10], and (ii) a protocol for physiotherapists to deliver subgroup-specific exercise therapy. This model was first tested for feasibility in a pilot-study in 50 patients with knee OA treated by physiotherapists in primary care [11]. Our pilot-study showed that the model is feasible and potentially (cost-)effective. Based on the findings from the pilot-study, the original model of five subgroups was adapted to a simplified model of three subgroups. First, a ‘low muscle strength subgroup’ that is comparable to the proposed ‘ageing phenotype’ [1] and expected to benefit most from exercise therapy targeting muscle strengthening, as the suggested most important working mechanism for exercise therapy [12, 13]. Second, a ‘high muscle strength subgroup’ that is comparable to the proposed ‘post-traumatic phenotype’ [1] and expected not to benefit from exercise therapy, as no clinical effects can be expected from further muscle strengthening [14]. Therefore, this subgroup should only receive a minimal intervention of education and advice [15]. Third, an ‘obesity subgroup’ that is comparable to the proposed ‘metabolic phenotype’ and expected to benefit most from exercise therapy targeting both muscle strengthening and aerobic capacity, supplemented with a weight loss intervention [15, 16]. In contrast to our proposed, subgroup-specific interventions, previous studies as well as current practice usually offers a standardized muscle strengthening program based on a ‘one size fits all’ approach [15]. We hypothesize that the three subgroups substantially differ in effects on pain and physical function of such a treatment, with large effects expected in the ‘low muscle strength subgroup’, medium effects in the ‘obesity subgroup’ and only small effects in the ‘high muscle strength subgroup’.

Our newly developed stratification algorithm could be an important instrument to identify homogeneous subgroups from the heterogeneous knee OA population. The accompanying treatment stratification is expected to play a key role in future optimization of knee OA care, by optimizing clinical effects and saving costs. Therefore, it is highly relevant to further explore the validity of this algorithm. In the present study, we will validate the construct of this algorithm, focusing on 3 research questions:

(i) Are the proportions of patients in each subgroup similar/consistent across cohorts (i.e., only minimal variations in subgroup proportions between cohorts)?
(ii) Are the characteristics of each of the subgroups in line with their proposed underlying phenotypes (i.e., the ‘high muscle strength subgroup’ representing a ‘post-traumatic phenotype’, the ‘low muscle strength subgroup’ representing an ‘age-induced phenotype’ and the ‘obesity subgroup’ representing a ‘metabolic phenotype’)?
(iii) Are the effects of usual exercise therapy in the 3 subgroups in line with the proposed effect sizes (i.e., large effect expected for the ‘low muscle strength subgroup’, medium effects for the ‘obesity subgroup’ and small effect for the ‘high muscle strength subgroup’)?

## Patients and methods

### Design

We used existing data of patients with knee OA from the following five studies:

One cross-sectional cohort:

(i) AMS-OA cohort (Netherlands) (cohort in a secondary care setting [5])

Four randomized controlled trial (RCT) cohorts, in which a 3-month, supervised exercise therapy program was provided:

(ii) STABILO-trial (Netherlands) [17]
(iii) NEXA-trial (Australia) [18]
(iv) CBT-trial (Australia) [19]
(v) VIDEX-trial (Netherlands) (De Zwart AH, Dekker J, Roorda LD, van der Esch M, Lips P, van Schoor NM, et al.: High-intensity resistance training and vitamin D supplementation for knee osteoarthritis: a randomized controlled trial, Under review).

For the present study, we used baseline data from the cross-sectional AMS-OA-cohort and data from both baseline and 3-month follow-up from the four trial cohorts. In all five studies, each patient provided written, informed consent according to the Declaration of Helsinki for participating in the particular study, and all studies were approved by the institutional Medical Ethical Review Board. In addition, we had formal data sharing agreements with institutions from each cohort. A full description of the inclusion and exclusion criteria of each of these five cohorts is provided in a Supplementary file. These criteria are highly comparable across cohorts, with clinical diagnosis of knee OA as the main inclusion criterion in all cohorts.

The AMS-OA-cohort is an ongoing cross-sectional cohort started from 2009, in which patients with knee and/or hip OA referred to an outpatient rehabilitation centre (Reade, Amsterdam) enrolled [5]. We used data from all patients (*n* = 553) who enrolled in the cohort until 2019 and were clinically diagnosed with knee OA, and excluded those patients that participated in the STABILO-trial [17] or VIDEX-trial (De Zwart AH, Dekker J, Roorda LD, van der Esch M, Lips P, van Schoor NM, et al.: High-intensity resistance training and vitamin D supplementation for knee osteoarthritis: a randomized controlled trial, Under review), as these patients also enrolled this cohort. The STABILO-trial was a two-arm RCT in 159 patients with knee OA, comparing a muscle strengthening exercise program with a muscle strengthening plus knee stabilization exercise program. Patient enrollment was between February 2009 and March 2011 [17]. The NEXA-trial was a two-arm RCT in 100 patients with medial compartment knee OA and varus malalignment, comparing a quadriceps strengthening exercise program with a neuromuscular exercise program. Patient enrollment was between July 2010 and June 2012 [18]. The CBT-trial was a three-arm RCT in 222 patients with clinically diagnosed knee OA, comparing a muscle strengthening exercise program, a pain coping skills training program and a combination of both. Patient enrollment was between May 2010 and January 2012 [19]. The VIDEX-trial was a two-arm RCT in 177 patients with clinically diagnosed knee OA, comparing a high-intensity resistance training (training intensity 70–80% of 1 repetition maximum) with a low-intensity exercise program (training intensity 40–50% 1 repetition maximum). Patient enrollment was between September 2014 to January 2018 (De Zwart AH, Dekker J, Roorda LD, van der Esch M, Lips P, van Schoor NM, et al.: High-intensity resistance training and vitamin D supplementation for knee osteoarthritis: a randomized controlled trial, Under review).

### Hypotheses

In line with the COnsensus-based Standards for the selection of health status Measurement INstruments (COSMIN) guideline [20], we formulated a priori hypotheses. A total of 63 hypotheses were therefore formulated, prior to our study, to test the construct validity of the stratification algorithm (see Table 1). The cut-off values used for accepting or refuting the hypotheses were decided by the authors, if possible based on existing or well-accepted values.

**Table 1:** Overview of a-priori hypotheses (*n* = 63) and scores to accept these hypotheses

RESEARCH QUESTION I: SIMILAR SUBGROUP PROPORTIONS	
Subgroup proportion in one cohort is similar to subgroup proportion in total sample	Deviation
***‘Low muscle strength subgroup’***	
AMS-OA [5] vs. total sample	−10, + 10%^1^
STABILO [17] vs. total sample	−10, + 10%^1^
NEXA [18] vs. total sample	−10, + 10%^1^
CBT [19] vs. total sample	−10, + 10%^1^
VIDEX (De Zwart AH, Dekker J, Roorda LD, van der Esch M, Lips P, van Schoor NM, et al.: High-intensity resistance training and vitamin D supplementation for knee osteoarthritis: a randomized controlled trial, Under review) vs. total sample	−10, + 10%^1^
***‘High muscle strength subgroup’***	
AMS-OA [5] vs. total sample	− 10, + 10%^1^
STABILO [17] vs. total sample	−10, + 10%^1^
NEXA [18] vs. total sample	−10, + 10%^1^
CBT [19] vs. total sample	−10, + 10%^1^
VIDEX (De Zwart AH, Dekker J, Roorda LD, van der Esch M, Lips P, van Schoor NM, et al.: High-intensity resistance training and vitamin D supplementation for knee osteoarthritis: a randomized controlled trial, Under review) vs. total sample	−10, + 10%^1^
***‘Obesity subgroup’***	
AMS-OA [5] vs. total sample	− 10, + 10%^1^
STABILO [17] vs. total sample	−10, + 10%^1^
NEXA [18] vs. total sample	−10, + 10%^1^
CBT [19] vs. total sample	−10, + 10%^1^
VIDEX (De Zwart AH, Dekker J, Roorda LD, van der Esch M, Lips P, van Schoor NM, et al.: High-intensity resistance training and vitamin D supplementation for knee osteoarthritis: a randomized controlled trial, Under review) vs. total sample	-10, + 10%^1^
**RESEARCH QUESTION 2: CHARACTERISTICS IN LINE WITH UNDERLYING PHENOTYPES**	
**Characteristic in one subgroup that is in line with proposed underlying phenotype is different from other subgroups**	**p-value**
***‘Low muscle strength subgroup’ (‘age-induced phenotype’)***	
Higher age, compared to:	
‘high muscle strength subgroup’	*P* < 0.05^2^
‘obesity subgroup’	*P* < 0.05^2^
Lower muscle strength, compared to:	
‘high muscle strength subgroup’	*P* < 0.05^2^
‘obesity subgroup’	*P* < 0.05^2^
‘***High muscle strength subgroup’ (‘post-traumatic phenotype’)***	
More history of knee surgery, compared to:	
‘low muscle strength subgroup’	*P* < 0.05^2^
‘obesity subgroup’	*P* < 0.05^2^
Higher muscle strength, compared to:	
‘low muscle strength subgroup’	*P* < 0.05^2^
‘obesity subgroup’	*P* < 0.05^2^
More males, compared to:	
‘low muscle strength subgroup’	*P* < 0.05^2^
‘obesity subgroup’	*P* < 0.05^2^
Younger age, compared to:	
‘low muscle strength subgroup’	*P* < 0.05^2^
‘obesity subgroup’	*P* < 0.05^2^
Higher K/L grade, compared to:	
‘low muscle strength subgroup’	*P* < 0.05^2^
‘obesity subgroup’	*P* < 0.05^2^
Less comorbidities, compared to:	
‘low muscle strength subgroup’	*P* < 0.05^2^
‘obesity subgroup’	*P* < 0.05^2^
Less severe knee pain, compared to:	
‘low muscle strength subgroup’	*P* < 0.05^2^
‘obesity subgroup’	*P* < 0.05^2^
Less impaired physical function, compared to:	
‘low muscle strength subgroup’	*P* < 0.05^2^
‘obesity subgroup’	*P* < 0.05^2^
***‘Obesity subgroup’ (‘metabolic phenotype’)***	
Higher BMI, compared to:	
‘high muscle strength subgroup’	*P* < 0.05^2^
‘low muscle strength subgroup’	*P* < 0.05^2^
More comorbidities, compared to:	
‘high muscle strength subgroup’	*P* < 0.05^2^
‘low muscle strength subgroup’	*P* < 0.05^2^
Lower muscle strength, compared to:	
‘high muscle strength subgroup’	*P* < 0.05^2^
‘low muscle strength subgroup’	*P* < 0.05^2^
More severe knee pain, compared to:	
‘high muscle strength subgroup’	*P* < 0.05^2^
‘low muscle strength subgroup’	*P* < 0.05^2^
More severe impaired physical function, compared to:	
‘high muscle strength subgroup’	*P* < 0.05^2^
‘low muscle strength subgroup’	*P* < 0.05^2^
**RESEARCH QUESTION 3: EFFECTS OF USUAL EXERCISE THERAPY IN LINE WITH HYPOTHESIZED EFFECTS**	
	**Effect size/ % with MIC**
***Large effects in ‘low muscle strength subgroup’***	
Large effect size on knee pain	0.8 ± 0.2
Majority with MIC on knee pain	> 67%
Large effect size on physical function	0.8 ± 0.2
Majority with MIC on physical function	> 67%
Large effect size on muscle strength	0.8 ± 0.2
Majority with MIC on muscle strength	< 67%
***Medium effects in ‘obesity subgroup’***	
Medium effect size on knee pain	0.5 ± 0.2
Half with MIC on knee pain	33–67%
Medium effect size on physical function	0.5 ± 0.2
Half with MIC on physical function	33–67%
Medium effect size on muscle strength	0.5 ± 0.2
Half with MIC on muscle strength	33–67%
***Small effects in ‘high muscle strength subgroup’***	
Small effect size on knee pain	0.2 ± 0.2
Minority with MIC on knee pain	< 33%
Small effect size on physical function	0.2 ± 0.2
Minority with MIC on physical function	< 33%
Small effect size on muscle strength	0.2 ± 0.2
Minority with MIC on muscle strength	< 33%

MIC = minimal important change; ^1^ difference in subgroup proportion (%)in one cohort compared to subgroup proportion in total sample; ^2^ p-value for differences between subgroups; ^3^ isokinetic knee extensor strength measure as outcome; ^4^ 30-s chair stand test as outcome; *significant finding in the opposite direction as expected, therefore hypothesis not accepted

We focused on the following three research questions:

1. Are the proportions of patients in each subgroup similar across cohorts?
2. Are the characteristics of each of the subgroups in line with their proposed underlying phenotypes?
3. Are the effects of usual exercise therapy in the 3 subgroups in line with the proposed effect sizes?

The first research question concerned 15 hypotheses in order to test whether proportions of each of the three subgroups were similar across the five cohorts. We compared subgroup proportions in each cohort with the average subgroup proportion for the total sample, in order to detect relevant deviations across cohorts. A relevant deviation in subgroup proportion was a priori defined as a deviation of more than 10%, when comparing the proportion of a subgroup in one cohort with the total sample.

The second research question concerned 30 hypotheses in order to test whether the subgroup characteristics were in line with their proposed underlying phenotype. Specifically, for the ‘low muscle strength subgroup’, we proposed that the underlying phenotype is the ‘age-induced phenotype’ as described by Bijlsma et al. [1], characterized by older age and a physically inactive lifestyle (i.e., low muscle strength). For the ‘high muscle strength subgroup’, we proposed that the underlying phenotype is the ‘post-traumatic phenotype’ as described by Bijlsma et al. [1], characterized by a history of knee trauma, a physically active lifestyle (i.e., high muscle strength), mostly males, young age, high grades of radiographic severity of knee OA, absence of comorbidities, and only mild levels of pain and impaired physical function. For the ‘obesity subgroup’, we proposed that the underlying phenotype is the ‘metabolic phenotype’ as described by Bijlsma et al. [1], characterized by high BMI, high number of comorbidities, physically inactive lifestyle (i.e., low muscle strength), and severe levels of pain and impaired physical function.

The third research question concerned 18 hypotheses to test whether the effects of usual exercise therapy on knee pain, physical function and muscle strength for each subgroup were in line with the expected effects. Specifically, we hypothesized that the effects of usual exercise therapy, which is predominantly standard strength training (as applied in the included trials), differ between subgroups. We expected large effects for the ‘low muscle strength subgroup’, due to the main focus of exercise therapy on muscle strengthening, which is proposed to be the most important working mechanism underlying the effects of exercise therapy on pain and physical function [12, 13]. We expected medium effects for the ‘obesity subgroup’, due to obesity-induced overloading of the knee hampering the ‘regular’ exercises to have optimal effects, as well as due to the necessity to lose weight, which cannot be achieved by usual exercise therapy [16]. We expected small effects for the ‘high muscle strength subgroup’, as this subgroup is unlikely to achieve any functional improvement by muscle strengthening above an already high level of strength [14].

### Measurements

First, baseline data from the following patient characteristics were used: age, sex, Kellgren/Lawrence (K/L) grade for radiographic severity of knee OA [21] (for knee with highest grade), history of knee surgery (only available in NEXA-trial [18] and CBT-trial [19]), and number of comorbidities (i.e., diseases other than knee OA) affecting daily life (i.e., Cumulative Illness Rating Scale (CIRS) [22] > 1) (only available in AMS-OA-cohort [5] and VIDEX-trial) (De Zwart AH, Dekker J, Roorda LD, van der Esch M, Lips P, van Schoor NM, et al.: High-intensity resistance training and vitamin D supplementation for knee osteoarthritis: a randomized controlled trial, Under review). Second, the following outcome measures at both baseline and 3-month follow-up were used: knee pain severity (assessed by a 0–10 scaled Numeric Rating Scale (NRS) in AMS-OA-cohort [5], STABILO-trial [17] and VIDEX-trial (De Zwart AH, Dekker J, Roorda LD, van der Esch M, Lips P, van Schoor NM, et al.: High-intensity resistance training and vitamin D supplementation for knee osteoarthritis: a randomized controlled trial, Under review), or a 0–100 scaled Visual Analogue Scale (VAS) in NEXA-trial [18] and CBT-trial [19]), which we re-scaled to 0–10) and physical function (assessed by 0–100 scaled Western Ontario and McMaster Universities Osteoarthritis Index (WOMAC) subscale physical function [23]). Third, data from the two stratification variables at baseline were used, namely BMI and quadriceps muscle strength (assessed by an isokinetic knee extension strength dynamometry [24] in AMS-OA-cohort [5], STABILO-trial [17] and VIDEX-trial (De Zwart AH, Dekker J, Roorda LD, van der Esch M, Lips P, van Schoor NM, et al.: High-intensity resistance training and vitamin D supplementation for knee osteoarthritis: a randomized controlled trial, Under review) (using the score of the ‘index knee’ (i.e., knee with diagnosed knee OA, or in case of bilateral knee OA, knee with highest K/L grade, or in case of similar grades, knee with lowest muscle strength score), or by the 30 s chair stand test (30s-CST) [25] in NEXA study [18] and CBT study [19]). The 3-month follow-up data on muscle strength was also used as an outcome measure.

Based on baseline scores of these two stratification variables, persons were allocated to one of the three subgroups as following (as shown by Fig. 1):

(i) persons with a BMI of 30 or higher (i.e. cut-off value for being obese) were allocated to the ‘obesity subgroup’;
(ii) from the remaining persons, those with (depending on which measure used in the cohort) or an isometric knee extensor strength score of 1.2 kg/m^2^ (i.e. threshold value above which an increase in strength is unlikely to result in any further functional improvement [14]) or a 30s-CST score of 12 repetitions or higher (i.e. cut-off value corresponding with patient acceptable symptom state in OA patients receiving exercise therapy after total joint arthroplasty [26], and corresponding with the normative value for community-dwelling older people of 60 years or older (females) or 65 years or older (males) [27]) were allocated to the ‘high muscle strength subgroup’;
(iii) all other persons were allocated to the ‘low muscle strength subgroup’.

### Statistical analysis

The hypotheses regarding the first research question on similar subgroup proportions were tested by comparing these subgroup proportions (%) in each cohort with the average subgroup proportion for the total sample. If the difference between the observed subgroup proportion in a cohort compared to the subgroup proportion in the total sample would not exceed the a priori formulated maximum deviation of ±10%,, the hypothesis was accepted.

The hypotheses regarding the second research question on underlying phenotypes were comparing the subgroups on a number of patient characteristics that are proposed to be indicative for the underlying phenotype of one of the subgroups (e.g., more male people in ‘high muscle strength subgroup’ compared to the other two subgroups). A *p*-value of 0.05 in a Chi-square test (for categorical variables) or an independent sample t-test (for other variables) was considered as a statistically significant difference between two subgroups, thereby accepting the hypothesis (if the difference was in line with the proposed underlying phenotype).

The hypotheses regarding the third research question on the effects of usual exercise therapy were tested by calculating within-group effect sizes (i.e., difference between baseline score and 3-month follow-up score, divided by baseline standard deviation) and responder rates for each subgroup separately and comparing these with the a priori hypothesized values. We hypothesized an effect size of 0.8 (large effect) in the ‘low muscle strength subgroup’, 0.5 (medium effect) in ‘obesity subgroup, and 0.2 (small effect) in ‘high muscle strength subgroup), and applied a maximal deviation of ±0.2 from the hypothesized effect sizes, to accept or refute our hypothesis. For responder rates, we calculated the number of participants reaching the currently accepted minimal important changes (MICs) for the NRS for knee pain severity (i.e., 15% and/or 1 point improvement [28]), WOMAC physical function subscale (i.e., 12% improvement [29]), isokinetic knee extension strength measurement (i.e., 30% improvement [30]) and 30s-CST (i.e., 2 repetitions improvement [31]). We hypothesized that in the ‘low muscle strength subgroup’, a majority (i.e., > 67%) reaches this MIC, in the ‘obesity subgroup’ around half of the patients (i.e., between 33 and 67%), and in the ‘high muscle strength subgroup only a minority (i.e., < 33%). For each of the 3 subgroups separately, an observed responder rate that is within this proposed range resulted in accepting the hypothesis.

All analyses were performed with SPSS version 26.

## Results

Data from a total of 1211 persons from the five studies were included in our study, of which 584 participated in an exercise therapy trial. As described in Tables 2, 553 persons were from the cross-sectional AMS-OA-cohort, 159 from the STABILO-trial, 100 from the NEXA-trial, 222 from the CBT-trial, and 177 from the VIDEX-trial. Based on an overall judgement of the clinical characteristics (e.g., pain, physical function, radiographic severity), the CBT-trial and AMS-OA-cohort seem to be the most severely affected cohorts, whereas the VIDEX-trial the least affected cohort.

**Table 2:** Characteristics in each cohort

	AMS-OA (5)	STABILO (17)	NEXA (18)	CBT (19)	VIDEX (20)	Total sample
N	553	159	100	222	177	1211
N with exercise therapy	0	159	100	148	177	584
**Demographics**						
Gender (female), n (%)	397 (72%)	97 (61%)	52 (52%)	133 (60%)	107 (61%)	786 (65%)
Age (years), mean ± SD	62.9 ± 9.4	61.9 ± 7.1	62.4 ± 7.3	63.4 ± 8.0	67.6 ± 5.8	63.5 ± 8.4
Radiographic severity:						
K&L grade 0/1, n (%)	185 (38%)	49 (31%)	0 (0%)	0 (0%)	61 (35%)	296 (26%)
K&L grade 2, n (%)	125 (25%)	44 (28%)	22 (22%)	90 (41%)	59 (33%)	340 (30%)
K&L grade 3, n (%)	97 (20%)	45 (28%)	43 (43%)	63 (28%)	29 (16%)	277 (24%)
K&L grade 4, n (%)	86 (17%)	21 (13%)	35 (35%)	69 (31%)	28 (16%)	239 (21%)
History of knee surgery, n (%)	n/a	n/a	49 (49%)	91 (41%)	n/a	140 (44%)
Nr. of comorbidities (CIRS ≥2):						
0, n (%)	259 (48%)	n/a	n/a	n/a	131 (76%)	390 (55%)
1, n (%)	152 (28%)				42 (24%)	194 (27%)
2, n [5]	73 (14%)				0 (0%)	73 (10%)
≥ 3, n (%)	58 (11%)				0 (0%)	58 (8%)
	**AMS-OA (5)**	**STABILO (14)**	**NEXA (15)**	**CBT (16)**	**VIDEX (17)**	**Total sample**
**Outcome variables**						
Knee pain (0–10, NRS/VAS), mean ± SD	5.8 ± 2.4	5.0 ± 2.1	5.4 ± 1.5	5.9 ± 1.3	4.7 ± 2.2	5.5 ± 2.1
Physical function (0–100, WOMAC), mean ± SD	47.1 ± 20.5	38.5 ± 18.0	40.0 ± 14.1	51.4 ± 10.7	30.7 ± 19.3	43.7 ± 19.2
**Stratification variables**						
Body mass index (kg/m^2^), mean ± SD	31.9 ± 6.7	29.0 ± 4.6	29.6 ± 4.1	31.1 ± 6.1	28.2 ± 4.4	30.6 ± 6.0
Quadriceps strength (Nm/kg), mean ± SD	0.84 ± 0.53	0.98 ± 0.51	n/a	n/a	1.12 ± 0.49	0.92 ± 0.53
30s-CST (repetitions), mean ± SD	n/a	n/a	10.7 ± 2.6	8.7 ± 2.7	n/a	9.3 ± 2.8

K/L = Kellgren & Lawrence; CIRS = Cumulative Illness Rating Score; WOMAC = Western Ontario and McMaster Universities Osteoarthritis Index; NRS = Numeric Rating Scale; VAS = Visual Analogue Scale; 30s-CST = 30 s chair stand test; n/a = not applicable (not assessed)

Table 3 shows the subgroup allocation in each of the five cohorts, to focus on our first research question on similar proportions. First, 35% of the total sample was allocated to the ‘low muscle strength subgroup’, ranging from 30% (AMS-OA-cohort) and 42% (CBT-trial). Second, 18% of the total sample was allocated to the ‘high muscle strength subgroup’, ranging across cohorts between 7% (CBT-trial) and 33% (VIDEX-trial). Third, 48% of the total sample was allocated to the ‘obesity subgroup’, which ranges between 29% (VIDEX-trial) and 56% (AMS-OA-cohort). As shown by Table 3, only three of the 15 subgroup proportions were outside the maximum difference of 10%, therefore 12 of the 15 hypotheses (80%) were accepted.

**Table 3:** Comparison of subgroup proportions across cohorts (research question 1; findings resulting in accepted hypotheses in bold)

	Low muscle strength subgroup		High muscle strength subgroup		Obesity subgroup	
	N (%)	Difference with total sample	N (%)	Difference with total sample	N (%)	Difference with total sample
**Total sample**	**421 (35%)**		**213 (18%)**		**547 (48%)**	
**AMS-OA (5)**	167 (30%)	**−5%**	79 (14%)	**−4%**	307 (56%)	**+ 8%**
**STABILO (17)**	60 (38%)	**+ 3%**	37 (23%)	**+ 5%**	62 (39%)	**−9%**
**NEXA (18)**	33 (33%)	**−2%**	23 (23%)	**+ 5%**	44 (44%)	**−4%**
**CBT (19)**	94 (42%)	**+ 7%**	16 (7%)	−11%	112 (51%)	**+ 2%**
**VIDEX (20)**	67 (38%)	**+ 3%**	58 (33%)	+ 15%	52 (29%)	−19%

In Table 4, baseline characteristics of each of the subgroups are displayed and compared with the other subgroups, for the second research question (characteristics in line with underlying phenotype). First, for the ‘low muscle strength subgroup’, both of the factors indicative of an ‘age-induced phenotype’ (i.e., older age and low muscle strength) were found to differ statistically significantly from the two other subgroups. Second, for the ‘high muscle strength subgroup’, 13 out of 16 subgroup comparisons (from 8 factors) aligned with the proposed ‘post-traumatic phenotype’, as they differed from the other two subgroups. The only two factors not found to statistically significantly differ were age (i.e., not lower but similar to ‘obesity subgroup’) and radiographic severity (i.e., not higher but similar or even lower to the other two subgroups), whereas the other six factors differed as expected. Third, for the ‘obesity subgroup’, all five factors that are indicative for a ‘metabolic phenotype’ (i.e., high BMI, large number of comorbidities, low muscle strength, high level of knee pain and low level of physical function) were found to be statistically significantly different from the other two subgroups, except for a similar level of knee pain compared to the ‘low muscle strength subgroup’. As shown by Tables 4, 26 out of 30 (87%) hypotheses were accepted.

**Table 4:** Comparison of observed subgroup characteristics that are in line with proposed underlying phenotype with other subgroups (research question 2; findings resulting in accepted hypotheses in bold)

	Low muscle strength subgroup (L)	High muscle strength subgroup (H)	Obesity subgroup (O)	Difference between L and H	Difference between L and O	Difference between H and O
**Baseline variables**						
Gender (female), n (%)	310 (74%)	88 (41%)	388 (67%)	***P*** **< 0.001**	*P* = 0.03	***P*** **< 0.001**
Age (years), mean ± SD	65.2 ± 8.4	62.3 ± 8.6	62.7 ± 8.3	***P*** **< 0.001**	***P*** **< 0.001**	*P* = 0.55
Radiographic severity:						
K&L grade 0/1, n (%)	95 (24%)	72 (35%)	128 (24%)	***P*** = 0.02*	*P* = 0.44	*P* < 0.001*
K&L grade 2, n (%)	131 (33%)	64 (31%)	145 (27%)			
K&L grade 3, n (%)	92 (23%)	48 (23%)	137 (25%)			
K&L grade 4, n (%)	83 (21%)	42 (12%)	132 (24%)			
Knee surgery, n (%)	51 (40%)	24 (63%)	65 (42%)	***P*** **= 0.01**	*P* = 0.80	***P*** **= 0.02**
Nr. of comorbidities:						
0, n (%)	142 (62%)	97 (71%)	151 (43%)	***P*** **= 0.02**	***P*** **< 0.001**	***P*** **< 0.001**
1, n (%)	61 (15%)	28 (21%)	105 (30%)			
2. n (%)	13 (3%)	8 (6%)	52 (15%)			
≥ 3, n (%)	14 (3)	3 (2%)	41 (12)			
**Outcome variables**						
Knee pain (0–10), mean ± SD	5.6 ± 2.0	4.7 ± 2.2	5.8 ± 2.1	***P*** **< 0.001**	*P* = 0.31	***P*** **< 0.001**
Physical function (WOMAC, 0–68), mean ± SD	43.6 ± 17.3	30.6 ± 18.2	48.7 ± 18.6	***P*** **< 0.001**	***P*** **< 0.001**	***P*** **< 0.001**
**Stratification variables**						
Body mass index (kg/m^2^), mean ± SD	26.4 ± 2.5	25.8 ± 2.4	35.5 ± 5.0	*P* < 0.01	***P*** **< 0.001**	***P*** **< 0.001**
Quad. strength (Nm/kg), mean ± SD	0.71 ± 0.31	1.62 ± 0.32	0.78 ± 0.48	***P*** **< 0.001**	***P*** **= 0.03**	***P*** **< 0.001**
30s-CST (repetitions), mean ± SD	8.5 ± 2.3	13.2 ± 1.2	9.0 ± 2.7	***P*** **< 0.001**	*P* = 0.15	***P*** **< 0.001**

K/L = Kellgren & Lawrence; CIRS = Cumulative Illness Rating Score; 30s-CST = 30 s chair stand test

* significant finding in the opposite direction as expected, therefore hypothesis not accepted

Table 5 shows the within-group effect sizes of exercise therapy and proportions reaching the MIC on knee pain, physical function and muscle strength, for each subgroup, for our third research question (effects of usual exercise therapy in line with hypothesis). The effects in the ‘low muscle strength subgroup’ on knee pain and physical function were as large as expected, but somewhat lower than expected for muscle strength. The effects in the ‘obesity subgroup’ were at least similarly large as the ‘low muscle strength subgroup’ for knee pain and physical function, which was not expected, while lower for quadriceps muscle strength, as expected. The effects in the ‘high muscle strength subgroup’, although lowest of the three subgroups, were not as low as we expected, except for muscle strength. As shown by Tables 5, 8 out of 18 (44%) hypotheses were accepted.

**Table 5:** Comparison of observed effects of usual exercise therapy for each subgroup with hypothesized effects (research question 3; findings resulting in accepted hypotheses in bold)

	Low muscle strength subgroup		Obesity subgroup		High muscle strength subgroup	
	Observed	Hypothesized	Observed	Hypothesized	Observed	Hypothesized
**Knee pain**						
Effect size^1^	**1.05**	**0.8 ± 0.2**	1.10	0.5 ± 0.2	0.82	0.2 ± 0.2
% persons with MIC^2^	**70%**	**> 67%**	72%	33–67%	66%	< 33%
**Physical function**						
Effect size^1^	**0.79**	**0.8 ± 0.2**	0.78	0.5 ± 0.2	0.49	0.2 ± 0.2
% persons with MIC^3^	**79%**	**> 67%**	76%	33–67%	76%	< 33%
**Quad. strength / 30s-CST**						
Effect size^1^	**0.74/ 0.73**	**0.8 ± 0.2**	**0.27/ 0.60**	**0.5 ± 0.2**	**0.19/ 0.32**	**0.2 ± 0.2**
% persons with MIC^4,5^	49% / 31%	> 67%	32% / 28%	33–67%	7% / 9%	< 33%

MIC = minimal important change. ^1^ Effect size (within-group) = change score within group / standard deviation at baseline; ^2^ MIC defined as improvement on NRS/VAS pain (0–100) ≥ 15% and/or ≥ 1 point [25]; ^3^MIC defined as improvement on WOMAC physical function (0–100) ≥ 12% [26]; ^4^ MIC defined as improvement on quadriceps strength ≥30% [27]; ^5^ MIC defined as improvement on 30s-CST ≥ 2 repetitions [28]

## Discussion

This study aimed to test the construct validity of our stratification algorithm that we recently developed to apply subgroup-specific treatments (‘stratified care’) in patients with knee OA. Based on a priori formulated hypotheses in three research questions, this study resulted in mixed findings regarding the construct validity of our algorithm. Therefore, the added value for current physiotherapy practice needs to be further examined, focusing on the question of whether matched treatments lead to better clinical outcomes.

First, applying our stratification algorithm in five different cohorts resulted in relatively consistent proportions of subgroups, especially in three of the five cohorts. One patient group (VIDEX-trial) seemed to be less severely affected compared to the other cohorts, which resulted in a higher proportion of the ‘high muscle strength subgroup’ and a lower proportion of the ‘obesity subgroup’. On the contrary, the patient group from the CBT-trial seemed to be more severely affected, resulting in a lower proportion of the ‘high muscle strength subgroup’ and a higher proportion in the ‘obesity subgroup’. It is possible that the minimal levels of knee pain and impaired physical function as inclusion criteria applied in this CBT-trial have played a role in this second finding. However, despite differences in inclusion criteria, setting and country, the proportions of the three subgroups were generally consistent, thereby confirmative for this aspect of construct validity.

Second, this study revealed that characteristics of each of the three subgroups can be considered in line with the proposed underlying phenotypes [1]. The ‘low muscle strength subgroup’ was, besides having weak muscles, relatively older, which is consistent with the ‘age-induced phenotype’. The ‘high muscle strength subgroup’ was consistent with the ‘post-traumatic phenotype’, as a majority had a history of knee surgery, were male, had a high level of muscle strength, no comorbidities and only mild levels of pain and impaired physical function. The ‘obesity subgroup’ was – besides having a BMI over 30 - found to have more comorbidities and more severe symptoms, which is indicative for a ‘metabolic phenotype’. These findings suggest that generally accepted knee OA phenotypes can be identified by stratifying using only two easily obtainable variables (i.e., BMI and quadriceps muscle strength). Conversely, the overlap in phenotype characteristics between subgroups should be acknowledged. For instance, history of knee surgery was present in 63% of the ‘high muscle strength subgroup’, but also in 40–42% of the other two subgroups. This implies that, if the stratification algorithm is being used in daily practice, the health care professional should bear in mind that treatment not only depends on subgroup allocation, but also on the individual’s characteristics, needs, and preferences.

Third, we aimed to confirm our hypothesis that the effects of usual exercise therapy targeting muscle strengthening differ across our subgroups, based on the presumption of muscle strengthening being the main working mechanism underlying the effects of exercise therapy [12, 13]. Although we did find subgroup differences as hypothesized in exercise effects for muscle strength (i.e., large for the ‘low muscle strength subgroup’, medium for the ‘obesity subgroup’, and only small for the ‘high muscle strength subgroup’), we did not find this for knee pain or physical function. Unexpectedly, in each of the three subgroups, medium to large effects for knee pain and physical function were found. These findings suggest that muscle strengthening is not the only working mechanism of exercise therapy, and possibly not the most important one either. Also other working mechanisms such as reduced knee joint inflammation, increased knee joint proprioception, increased joint mobility and improved psychological factors seem to play a role. In addition, a proportion of the effect can possibly be attributed to the patient education targeting self-management that was provided alongside the exercise therapy, or to a more generalized effect of the physiotherapy (e.g. supervision and attention from the physiotherapist). This also implies that our two subgroup factors (i.e., upper leg muscle strength and BMI) may be less important as an effect mediator and/or effect modifier for exercise therapy than expected. Another explanation could be that, although a standardized, protocolized exercise therapy intervention has been described for the included RCTs, the participating physiotherapist may have provided a tailored, individualized treatment, resulting in a ‘stratified’ rather than a ‘non-stratified’ approach. For the ‘obesity subgroup’, the large treatment effects are even more surprising as almost none of the patients lost weight (i.e., 1% of the ‘obesity subgroup’ reached the MIC of 10% weight loss [30]). Based on this finding, we propose that when combining an exercise therapy intervention with a successful weight loss intervention, even larger effects can be reached. This could have substantial impact on both the level of the patient and society, as obesity is highly prevalent among persons with knee OA. Moreover, weight loss – with or without exercise – is expected to play a role in delaying structural progression of knee OA, next to improving clinical outcomes [32]. So far, only limited evidence is available on the effect of such a combined intervention in obese persons with knee OA [33–35], but is currently being tested in multiple trials, including our OCTOPuS-trial [15].

A few limitations in our study design should be noted. First, we combined multiple trial cohorts with multiple exercise groups within trials, so different exercise regimes were grouped together in our analyses. However, this is unlikely to have influenced our study findings, as each of the included exercise programs were highly comparable, with strength training as their main component. Second, three of the five included cohorts were from the same institute (AMS-OA, STABILO and VIDEX), whereas we would have preferred including cohorts from more different institutes to increase the generalizability of our study findings. Third, the decision to accept or refute the hypotheses were based on arbitrary, although mostly generally accepted cut-off points in subgroup proportions, *p*-values, effect sizes and MICs. If other cut-off points had been chosen, our conclusions could have been different. Fourth, we should emphasize that other subgroups may exist, alongside our three subgroups. In a recent review study, Dellisola et al. [3] proposed two additional phenotypes that might be of clinical relevance, namely a ‘malaligned phenotype’ (i.e., persons with varus or valgus knee alignment, in which biomechanical interventions like bracing might be necessary), and a ‘chronic pain phenotype’ (i.e., persons with psychological comorbidities (e.g. depressive mood), in which additional psychological or pain management interventions are needed). This latest subgroup was included in our original model that was tested for its feasibility [11], but because of its low prevalence in primary care physiotherapy, we decided to remove this subgroup from our model. Therefore, we should bear in mind that our stratification algorithm may not be useful for every person with knee OA. Finally, we would like to mention that the method of formulating and testing a large number of a priori hypotheses is the recommended method to test construct-validity [20]. As stated in this COSMIN-guideline, ‘*the more hypotheses are being tested on whether the data correspond to a priori formulated hypotheses, the more evidence is gathered for construct validity.’* Therefore*,* the large number of a priori hypotheses is a strength of our study design.

To conclude, we found mixed results regarding the construct validity of our stratification algorithm. On the one hand, it is a valid instrument to consistently allocate patients with knee OA into subgroups that aligned our hypotheses. On the other hand, in contrast to our hypotheses, these subgroups did not differ substantially in effects of usual exercise therapy. An ongoing RCT (OCTOPuS-study [15]) will demonstrate whether our stratification algorithm together with subgroup-specific exercise therapy improves clinical and economic outcomes, thereby having added value for clinical practice.

## Supplementary Information

**Additional file 1.**

## Data Availability

The data that support the findings of this study are available from the five studies (Reade, University of Melbourne) but restrictions apply to the availability of these data, which were used under license for the current study, and so are not publicly available. Data are however available from the authors of the original studies upon reasonable request and with permission of Reade and/or University of Melbourne.

## Abbreviations

30s-CST: 30 s Chair Stand Test
BMI: Body Mass Index
CIRS: Cumulative Illness Rating Scale
COSMIN: COnsensus-based Standards for the selection of health status Measurement INstruments
K/L: Kellgren/Lawrence
MIC: Minimal Important Change
NRS: Numeric Rating Scale
OA: OsteoArthritis
RCT: Randomized Controlled Trial
VAS: Visual Analogue Scale
WOMAC: Western Ontario and McMaster Universities Osteoarthritis Index
