# Supplementary material for: Construct validity of the OCTOPuS stratification algorithm for allocating patients with knee osteoarthritis into subgroups
Source: BMC Musculoskelet Disord. 2021 Jul 21;22:633. doi: 10.1186/s12891-021-04485-1 (PMC8296670; doi:10.1186/s12891-021-04485-1)
Supplement: Supplementary file 1 — Additional file 1. [file 12891_2021_4485_MOESM1_ESM.docx]

**SUPPLEMENTARY FILE: INCLUSION AND EXCLUSION CRITERIA OF INCLUDED STUDIES**

| **AMS-OA (5)** | **STABILO (17)** | **NEXA (18)** | **CBT (19)** | **VIDEX (20)** |
| --- | --- | --- | --- | --- |
| **Inclusion criteria** |  |  |  |  |
| - Referred to rehabilitation centre  - Diagnosis of knee OA according to clinical American College of Rheumatology (ACR) criteria (1) | - Diagnosis of knee OA according to clinical American College of Rheumatology (ACR) criteria (1)  - Age between 40 and 75 years  - Presence of self-reported and/or biomechanically assessed knee instability | - Average knee pain over the past week of >25/100 on VAS  - Pain/tenderness predominantly over the medial knee region  - Radiographic knee OA in medial compartment (K/L grade ≥2 (2))  - Varus malalignment (3,4) | - Referred to rehabilitation centre  - Diagnosis of knee OA according to clinical American College of Rheumatology (ACR) criteria (1)  - Age ≥50 years  - Knee pain for ≥3 months  - Average pain during previous week ≥40/100 on VAS  - At least moderate difficulty with daily activities (WOMAC physical function subscale ≥ 25/68) | - Referred to rehabilitation centre  - Diagnosis of knee OA according to clinical American College of Rheumatology (ACR) criteria (1)  - Age between 55 and 80 years  - Vitamin D level (25(OH)D level) > 15 nmol/L |
| **Exclusion criteria** |  |  |  |  |
| - Total knee replacement  - Rheumatoid arthritis, or any other form of inflammatory arthritis (i.e., crystal arthropathy or septic arthritis) | - Total knee replacement received or planned in near future  - Rheumatoid arthritis, or any other form of inflammatory arthritis (i.e., crystal arthropathy or septic arthritis)  - Presence of comorbidity resulting in severe activity limitations  - Severe knee pain (i.e., NRS>8), - Insufficient comprehension of Dutch language  - Inability to be scheduled for therapy  - Unwillingness to give informed consent | - Knee surgery or intraarticular corticosteroid injection within 6 months  - Current or past (within 4 weeks) oral corticosteroid treatment  - Systemic arthritic conditions  - Prior hip or knee joint replacement or tibial osteotomy surgery  - Other nonpharmacologic treatment within the past 6 months  - Body mass index of 36 kg/m2 | - Systemic arthritic conditions such as rheumatoid arthritis  - Medical condition precluding safe exercise such as uncontrolled hypertension or heart condition  - Self-reported history of serious mental illness, such as schizophrenia, or self-reported diagnosis of current clinical depression  - Neurological condition such as Parkinson’s disease, multiple sclerosis or stroke  - Knee surgery including arthroscopy within the past 6 months or total joint replacement  - Awaiting or planning any back or lower limb surgery within the next 12 months  - Current or past (within 3 months) oral or intra-articular corticosteroid use  - Physiotherapy, chiropractic or acupuncture treatment or exercises specifically for the knee within the past 6 months  - Walking exercise for >30 minutes continuously daily  - Participating in a regular (more than twice a week) structured and/or supervised exercise program such as attending exercise classes in a gym or use of a personal trainer  - Participating in or previous participation in a formal PCST program  - Inability to walk unaided  - Inadequate written and spoken English  - Inability to comply with the study protocol such as inability to attend physical therapy sessions or attend assessment appointments at the University | - Other forms of arthritis than OA (e.g. crystal arthropathy, septic arthritis, spondylarthropathy)  - Absolute contraindication for exercise therapy or resistance training  - Inability to perform strength training program due to severe co-morbidity  - Psychoneuroticism (SCL90 > 200)  - Total knee arthroplasty or scheduled for upcoming year  - Supervised strength training program >30 minutes/week in past 3 months  - Use of vitamin D supplements >800 IU  - Diagnosed with hypercalcemia, hyperparathyroidism or sarcoidosis  - Living in a nursing home  - Inability to be scheduled for therapy  - Insufficient comprehension of Dutch language  - No informed consent |

- - 1. Altman R, Asch E, Bloch D, Bole G, Borenstein D, Brandt K, et al. Development of criteria for the classification and reporting of osteoarthritis: classification of osteoarthritis of the knee. Arthritis Rheum 1986;29:1039e49.
    2. Kellgren JH, Lawrence JS. Radiological assessment of osteoarthrosis. Ann Rheum Dis 1957;16:494–502.
    3. Kraus VB, Vail TP, Worrell T, McDaniel G. A comparative assessment of alignment angle of the knee by radiographic and physical examination methods. Arthritis Rheum 2005;52:1730–5.
    4. Hinman RS, May RL, Crossley KM. Is there an alternative to the full-leg radiograph for determining knee joint alignment in osteoarthritis? Arthritis Rheum 2006;55:306–13.

K/L = Kellgren/Lawrence; NRS = numeric rating scale; PCST = pain coping skills training; SCL90 = Symptom CheckList 90; VAS = visual analogue scale; WOMAC = Western Ontario and McMaster Universities Osteoarthritis Index
